# Eliminating the high-risk CTV1 margin in DAHANCA oropharyngeal radiotherapy: Dosimetric impact on dysphagia and organ-at-risk doses

**DOI:** 10.2340/1651-226X.2025.44049

**Published:** 2025-09-11

**Authors:** Christian Rønn Hansen, Anders S. Bertelsen, Irene Hazell, Sarah W. Stougaard, Jørgen Johansen, Jens Overgaard, Jesper Grau Eriksen, Ruta Zukauskaite

**Affiliations:** a Laboratory of Radiation Physics, Odense University Hospital, Odense, Denmark; b Danish Centre for Particle Therapy, Aarhus University Hospital, Aarhus, Denmark; c Department of Clinical Research, University of Southern Denmark, Odense, Denmark; d Department of Oncology, Odense University Hospital, Odense, Denmark; e Department of Experimental Clinical Oncology, Aarhus University Hospital, Aarhus, Denmark; f Department of Oncology, Aarhus University Hospital, Aarhus, Denmark

**Keywords:** Head and neck cancer, radiotherapy, clinical target volume, treatment planning, margin

## Abstract

**Background and purpose:**

Radiotherapy for head and neck cancer must balance tumour control with late toxicities such as dysphagia and xerostomia. Recent retrospective studies suggest that the margin from the gross tumour volume (GTV) to the high-dose clinical target volume (CTV1) may not be critical for local control, while larger irradiated volumes increase the risk of toxicity. The study quantifies potential reductions in dose to organs at risk (OARs) and predicted dysphagia risk when the standard 5 mm GTV-to-CTV1 margin is eliminated in oropharyngeal cancer.

**Patient/material and methods:**

Retrospectively 30 oropharyngeal cancer patients treated consecutively during 2023 according to the DAHANCA guidelines (5 mm GTV-to-CTV1 margin) were selected. For each patient, a standard plan and a modified experimental plan (CTV1 = GTV, and CTV2 reduced by 5 mm accordingly) were generated using Pinnacle3 Auto-Planning. All plans met the DAHANCA target coverage and OAR dose constraints. Dose-volume data for relevant OARs were extracted and compared in MATLAB. Normal tissue complication probability (NTCP) model for dysphagia was applied.

**Results:**

Margin elimination reduced high-dose CTV volumes by 70%, yielding significant dose reductions to multiple OARs. Mean doses to the upper/middle pharyngeal constrictors decreased by around 4–5 Gy (*p* < 0.001) and to the contralateral submandibular gland by ~5 Gy (*p* < 0.001). These dosimetric gains correspond to an estimated median ΔNTCP of 6.0% of late grade ≥ 2 dysphagia. Target coverage and conformity were maintained in all plans.

**Interpretation:**

Omitting the high-risk CTV margin can substantially reduce the dose to dysphagia-associated OAR without compromising target coverage. This approach shows promise for improving patient-reported swallowing outcomes and warrants clinical evaluation.

## Introduction

Radiotherapy (RT) for head and neck cancer achieves high cure rates but can cause debilitating late toxicities. Dysphagia, difficulty swallowing, is a particularly impactful toxicity, negatively affecting the quality of life (QoL) [1]. Indeed, one-third of long-term survivors of head and/neck cancer report persistent swallowing difficulties, underscoring the need for RT strategies that spare swallowing structures [2]. To mitigate such effects, modern techniques like intensity-modulated RT (IMRT) allow dysphagia-optimised planning, reducing the dose to pharyngeal constrictor muscles (PCM) and other dysphagia-related structures [3]. The phase III DARS trial recently demonstrated that such swallow-sparing IMRT significantly improves patient-reported swallowing function at 12 months post-RT, with treated patients reporting better MD Anderson Dysphagia Inventory (MDADI) scores and fewer severe swallowing complications than those receiving standard plans.

Another potential avenue to reduce normal tissue toxicity is to minimise the irradiated tissue volume. In definitive head and neck RT, clinical target volumes (CTVs) are typically generated by geometric expansion of the gross tumour volume (GTV) to account for subclinical disease extension [4]. However, growing evidence suggests that large GTV-to-CTV margins may be unnecessary for achieving local tumour control in certain scenarios [5, 6]. A nationwide Danish analysis compared outcomes before versus after the 2013 adoption of a uniform 5 mm GTV–CTV1 margin (reduced from historically larger margins ~10 mm). The 3-year local control rates were equivalent (84% vs. 87%, *p* = 0.06) despite the smaller CTVs, indicating no detriment in tumour control. Most local recurrences occurred within the high-dose CTV in both eras, suggesting that microscopic spread beyond the GTV was effectively covered even with the reduced margin. Likewise, a multi-institutional study of p16-positive oropharyngeal cancers reported no increase in local failures among patients where no GTV expansion was applied compared to those with standard margins [6, 7].

In contrast, larger irradiated volumes have been correlated with higher normal tissue toxicity. Retrospective data indicate that a greater GTV–CTV1 margin contributes to increased rates of late dysphagia. For example, an analysis of 1,706 head and neck cancer patients treated in 2010–2015 found that patients treated with large high-risk CTV margins experienced significantly more severe dysphagia than those with smaller margins (52% vs. 48% grade 2–4 dysphagia; *p* = 0.003). In that cohort, tumour volume (GTV size) was the dominant geometric predictor of dysphagia risk, followed by the GTV–CTV1 expansion margin. These findings imply that shrinking the high-dose target volume could meaningfully reduce the burden of treatment-related swallowing dysfunction, provided local control is not compromised [6, 8, 9].

Building on this rationale, we hypothesised that completely eliminating the GTV-to-CTV1 margin, that is treating only the GTV as the high-dose volume, could further spare normal tissues and reduce toxicity compared to the standard 5 mm margin approach. This study aimed to quantify the dosimetric impact of a no-margin (GTV = CTV1) planning technique on organs at risk (OAR) in head and neck RT.

## Patients/material and methods

The planning study involved 30 consecutively treated patients with oropharyngeal squamous cell carcinoma treated with definitive chemoradiotherapy in 2023 at a single institution. All patients had bilateral neck irradiation indicated, either due to nodal involvement or midline primary location and were treated according to the national DAHANCA 2020 RT guidelines [10, 11]. Key inclusion criteria for this study were: primary oropharyngeal tumour, curative intent RT with bilateral neck targets, and availability of complete planning data. Patients with prior head/neck RT or postoperative RT were excluded to maintain a homogeneous cohort of intact primary treatments. The cohort included p16-positive and p16-negative oropharynx tumours (tonsil, base of tongue, etc.), reflecting the typical case mix.

The study follows the internationally accepted guidelines for treatment planning study, RATING, in the attempt to make the results as generalisable and objective as possible [12].

As per clinical the DAHANCA protocol, the GTV included the primary tumour as delineated on planning CT fused with diagnostic MRI and PET, and involved lymph nodes, if any, for the nodal GTV. The clinical standard plan ‘Clinical’ used a CTV1 defined as GTV plus a uniform 5 mm margin corrected for natural anatomical boundaries, representing the high-dose volume, and prescribed 66 Gy in 33 fractions for GTV < 4 cm or 68 Gy in 34 fractions for GTV > 4 cm. The CTV2 (60 Gy dose prescription) encompassed potential microscopic disease regions and was generated from CT1 + 5 mm, per DAHANCA guidelines. The elective target volumes, called CTV3 (50 Gy dose prescription) followed the international recommendation from Gregoire et al. [13]. For the modified plan ‘Margin’, the CTV1 margin was 0 mm, that is set CTV1 equal to the GTV, and adjusted the intermediate-risk volume such that CTV2 = (new) CTV1 + 5 mm. In practice, this meant that both the original CTV1 and CTV2 volumes were contracted by 5 mm in all directions. The new CTV2 thus covered approximately the same volume as the CTV1 in the clinical plan. All other aspects of target delineation (e.g. nodal levels included) remained the same. For all plans, a planning target volume (PTV) margin of 3 mm was applied around each CTV to account for setup uncertainty and patient motion, in line with the local clinical protocol [14].

Paired treatment plans were created for each patient: one adhering to the standard target volumes (clinical plan) and one with margin-reduced target volumes (experimental plan). To ensure comparability, we employed the Pinnacle^3^ (Philips) Auto-Planning module to generate both plans with minimal human bias. Each patient’s two plans used the same beam arrangement of one arc Volumetric Modulated Arc Therapy (VMAT) and optimisation template. The only intentional difference was the CTV definitions. The auto-planner was configured with institutional dose objectives based on DAHANCA guidelines, including target coverage requirements and dose constraints for OAR. After auto-planning, each plan was reviewed, and, if necessary, minor manual fine-tuning was performed to meet all clinical criteria, for example, hotspot smoothing or slight adjustment of target coverage optimisation. Importantly, both the clinical and margin-less plans were required to satisfy the full set of target and OAR dose constraints specified by guidelines [12]. This automated planning approach ensured that any dosimetric differences between the two plans could be attributed to the target volume modification alone rather than variability in the planner technique. All optimised plans were calculated for delivery on a linear accelerator with 6 MV photons, with daily image guidance employed clinically.

Plans were exported using DICOM and analysed using in-house MATLAB. Dose-volume histograms (DVHs) were generated for all relevant OARs. The high-dose target volume sizes (CTV1) were also compared between plans to quantify the volume reduction achieved by omitting the margin. Additionally, target coverage metrics (e.g. PTV D98%, D2%) were evaluated to ensure that the modified plans maintained adequate tumour dose coverage. All dose metrics were averaged over the 30 patients for each plan type.

A paired Wilcoxon signed-rank test was used to assess differences in OAR mean doses between the clinical versus margin-less plans for each structure. A *p* < 0.05 was considered statistically significant. To visualise the potential dose ranges of a significant difference, a p-value curve was calculated. Although this calculation was not statistically robust due to the highly correlated DVH metrics, it provided a useful tool to evaluate where the DVH difference was substantial [15].

A normal tissue complication probability (NTCP) model for late dysphagia was applied to translate dose differences into an estimate of clinical impact. Specifically, the logistic regression model adapted from Van den Bosch et al. estimates the probability of physician-rated late grade ≥ 2 swallowing dysfunction. The model uses baseline dysphagia, tumour site and the mean dose to the pharyngeal constrictors (PCM) and oral cavity [16]. The reported NTCP was calculated using a baseline of 0, and compared to a baseline of 1 for the discussion.

The study was conducted under the DAHANCA FORWARD project approval from the national ethical committee (NVK 2401283). All patient data were fully anonymised, and this was a planning study with no intervention affecting patient care.

## Results

Eliminating the 5 mm high-risk CTV1 margin (CTV1 = GTV) resulted in a substantially smaller CTV1 volume. The median CTV1 was reduced by 70%, from a median of 27.1 [interquartile range (IQR) 16–41] cm³ in the clinical plans to 8.0 [4–14] cm³ in the modified plans. This reflects the omission of the 5 mm peripheral margin around the tumour. The CTV2 in modified plans was also reduced compared to clinical plans, as it was contracted by 5 mm, from 56.4 [35–77] cm³ to 27.1 [16–41] cm³. The CTV3 was moderately reduced since the elective lymph node levels were the same; only the reduced CTV2 was the cause of this. The CTV3 volume went from 108.2 [90–132] cm^3^ to 78.5 [64–102] cm^3^. The CTV and PTV volumes are shown in Table 1. Despite these volume reductions, target coverage was equivalent between plan types. By design, all plans achieved the prescription dose to ≥ 98% of the PTVs volume received more than 95% dose prescription. DVH comparison can be seen in Supplementary Figures 1–3. For the DVH of CTV3 the reduced CTV1 and CTV2 volumes make the overdosage significantly smaller. The average dose to the CTV3 is reduced from 59.0 to 57.2 Gy, corresponding to the area between the two DVHs in Supplementary Figure 3.

**Table 1:** Median and interquartile range (IQR) of target volumes (cm³) for each target volume in the standard and margin-less CTV1 plans. The margin-less approach results in consistently lower volumes for CTV and PTV regions.

Target	Standard plan		Margin-less plan	
	Median	IQR	Median	IQR
CTV1	27.1	16.0–41.0	8.0	4.0–13.5
CTV2	56.4	34.8–77.0	27.1	16.0–41.0
CTV3	108.2	89.5–131.5	78.5	63.8–102.0
PTV1	48.4	31.3–67.9	19.5	11.0–30.4
PTV2	88.6	55.3–113.7	48.3	31.3–41.0
PTV3	197	156.0–220.8	165.4	131.7–200.7

PTV: planning target volume.

The margin-less plan approach resulted in consistent dose reductions in surrounding normal structures, most notably those adjacent to the primary tumour. Table 2 summarises the mean dose to each OAR in the clinical versus modified plans. Among swallowing-related structures, the dose to the two cranial PCMs was subject to a significant dose reduction. The inferior PCM had a lower baseline dose (~35 Gy) and showed no significant change, as this muscle is usually partly outside the high-dose field for oropharynx cancers. The oral cavity, which can be an unsparing bystander in large fields, had its mean dose reduced by ~5.4 Gy, reflecting less dose to the mouth when fields are tightened to the tumour.

**Table 2:** Mean and standard deviation (STD) of organ-at-risk (OAR) doses for the standard and margin-less CTV1 plans. The margin-less approach results in statistically significant dose reductions in multiple OARs, including the oral cavity, pharyngeal constrictor muscles (PCM), larynx, and salivary glands. The absolute dose difference (mean_standard − mean_marginless), corresponding OAR volumes (in cm³), and p-values from paired comparisons are provided.

OAR	Standard plan		Margin-less plan		Difference	Volume	*p*
	Mean	STD	Mean	STD			
OralCavity	37.1	18.2	31.8	17.0	5.4	102.6	< 0.001
PCM up	47.7	14.0	43.8	13.9	3.9	5.6	< 0.001
PCM mid	51.8	9.1	46.8	9.8	5.0	3.9	< 0.001
PCM low	35.6	6.3	35.1	5.6	0.4	2.2	0.29
Esophagus	15.0	14.6	15.3	14.8	-0.3	17.3	0.72
LarynxG	26.8	5.8	25.0	6.0	1.7	3.1	0.024
LarynxSG	40.8	12.8	35.3	12.5	5.5	13.8	< 0.001
Thyroid	33.3	13.7	33.8	13.3	-0.5	19.0	0.06
Parotid R	23.8	17.5	21.5	16.4	2.3	30.3	0.012
Parotid L	22.2	18.2	20.1	17.0	2.1	30.3	0.012
Subman R	52.1	6.6	47.0	8.2	5.1	9.3	< 0.001
Subman L	50.1	6.3	45.0	8.2	5.1	8.5	< 0.001

Dose to the salivary glands was also reduced with the margin-less plans. Notably, the submandibular glands had mean doses reduced by about 5 Gy on each side. These are substantial reductions of around an absolute 10%, bringing the means closer to the 35 Gy threshold associated with improved salivary function. The parotid glands also benefited, while the parotid dose change in absolute terms were smaller, relative terms (~9% reduction) could still be meaningful for xerostomia risk, especially if it helps keep the mean dose < 26 Gy in an at-risk gland.

**Figure 1:**
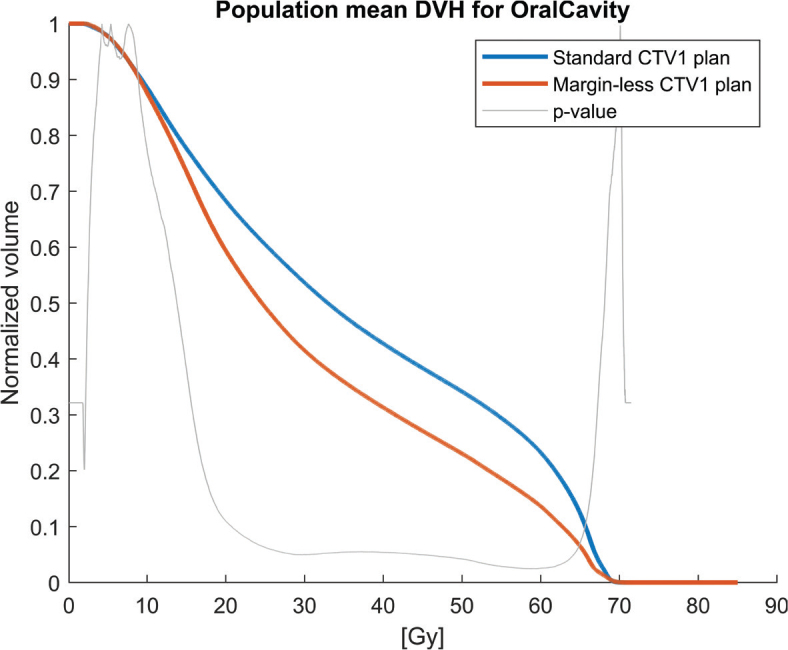
Population mean dose-volume histogram (DVH) for the Oral Cavity comparing standard CTV1 plans (blue) and margin-less CTV1 plans (red). The margin-less approach results in a consistent reduction in dose across the volume of the oral cavity. The grey curve indicates the dose-bin-wise p-values of the dose difference, demonstrating statistically significant dose sparing over a broad range of the dose distribution.

**Figure 2:**
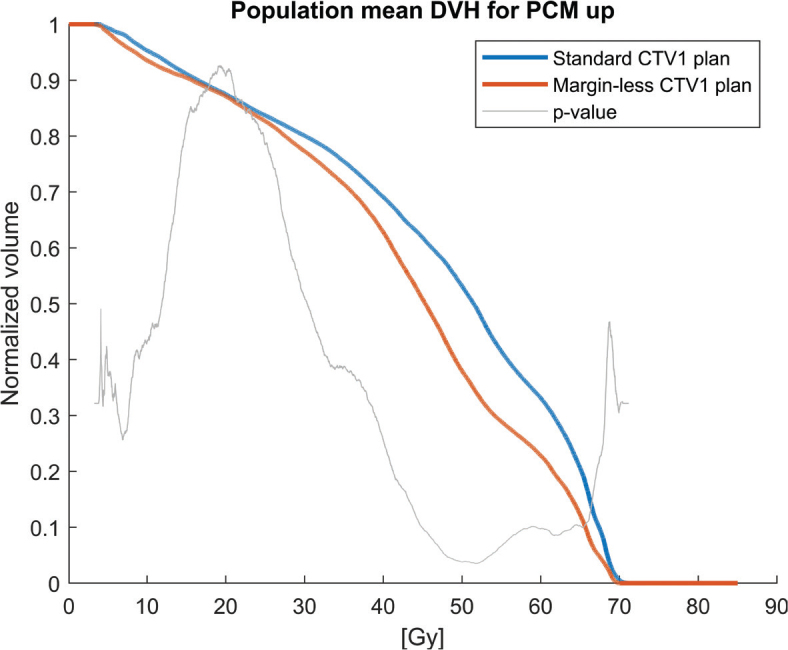
Population mean dose-volume histogram (DVH) for the upper pharyngeal constrictor muscle (PCM up) for standard CTV1 plans (blue) versus margin-less CTV1 plans (red). The margin-less plans result in a consistent dose reduction throughout the volume. The dose-bin-wise p-value curve (grey) shows significant differences across a broad mid-dose range.

**Figure 3:**
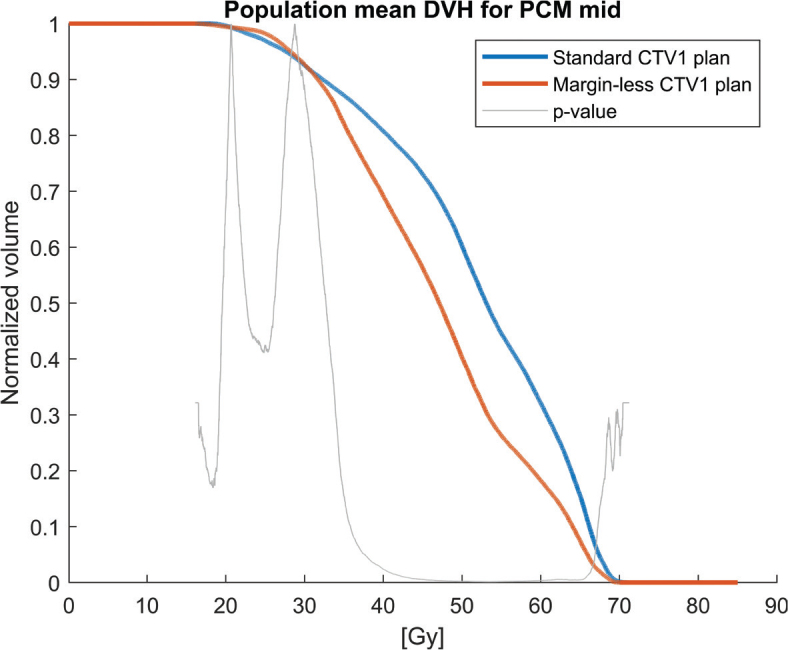
Population mean dose-volume histogram (DVH) for the mid pharyngeal constrictor muscle (PCM mid) comparing standard CTV1 plans (blue) and margin-less CTV1 plans (red). The margin-less approach leads to a notable reduction in dose across most of the DVH curve. The grey line indicates the dose-bin-wise p-values, demonstrating statistically significant differences in favour of the margin-less strategy across several dose levels.

**Figure 4:**
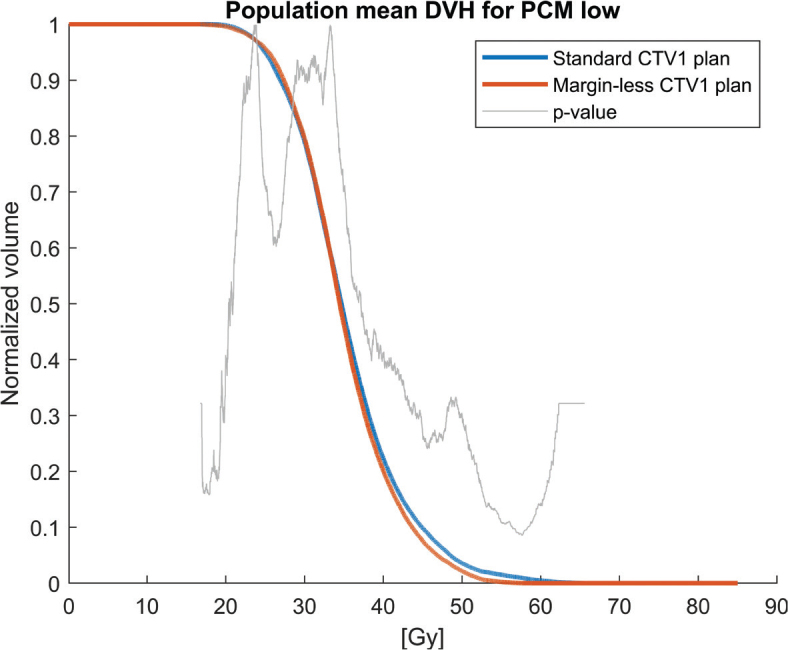
Population mean dose-volume histogram (DVH) for the lower pharyngeal constrictor muscle (PCM low), comparing standard (blue) and margin-less (red) CTV1 plans. Although differences are more modest than in other PCM regions, the margin-less approach still provides slight dose reductions. The dose-bin-wise p-values (grey) indicate statistically significant differences in selected dose intervals.

Population mean DVHs for the PCMs and oral cavity across the 30 patients, comparing the clinical plan (5 mm margin, blue solid lines) to the margin-less plan (0 mm margin, red solid lines) are shown in Figure 1–4. The margin-less plans consistently shift the DVH to the left, indicating lower volumes of the organ receiving high doses. All patients show improvement in constrictor sparing without loss of target coverage.

Almost all observed dose differences for OARs adjacent to the primary tumour were statistically significant in favour of the margin-less approach (Table 2). The only exceptions were structures already receiving low doses in the clinical plan, such as the inferior PCM, oesophagus, thyroid gland, and caudal from the primary oropharynx target.

Representative population DVHs for salivary glands, oesophagus, thyroid, glottic larynx, and supraglottic larynx are shown in Supplementary Figures 4–10. The margin-less plan DVH shows a clear reduction in high and intermediate doses to the submandibular gland.

The clinical significance of the observed dose differences was estimated using published NTCP models [16]. For physician-rated late dysphagia, the probability of developing symptomatic swallowing difficulties is strongly associated with the mean dose to the pharyngeal constrictors and the oral cavity. Using the NTCP model, the risk of late grade ≥ 2 dysphagia was 25.8% [IQR 19–38%] in the clinical plans and 19.6% [16–26%] in the margin-less plans, which translates into a median ΔNTCP of 6.0% [3.4–8.5%], highly significant (*p* < 0.001), all with baseline at 0. For a baseline of 1, the median ΔNTCP increased slightly to 7.4% [4.7–12%]. The NTCP model for physician-grated xerostomia ≥ 2 grade was 18.5% [16.7–19.5%] in the clinical plans and 17.2% [16.6–18.5%] in the margin-less plans, which translates into a median ΔNTCP of 0.4% [0.0–1.5%] statistically significantly (*p* = 0.001); however, presumably of negligible clinical effect.

## Discussion and conclusion

The dosimetric consequences of a zero mm GTV to CTV1 margin strategy in oropharyngeal RT, markedly reduce radiation dose to OAR associated with swallowing and salivation. Using an automated planning system to ensure consistency, we found that margin-less plans achieved equivalent target coverage while significantly sparing normal tissues. The magnitude of dose reductions in a range likely to decrease toxicity. These findings support the initial hypothesis that if local control is maintained, minimising high-dose target volume is a viable avenue to improve the therapeutic ratio in head and neck cancer.

The results build upon a growing body of evidence questioning the necessity of generous CTV margins in the era of improved medical imaging. Zukauskaite et al. reported that standardising to a 5 mm GTV margin from earlier, larger margins did not compromise local control in a national cohort [5, 6]. However, a recent study observed that patients treated with larger margins had worse swallowing outcomes [9]. These real-world data align with the dosimetric observation that larger high-dose volumes increase the dose to critical structures and, consequently, the risk of toxicity. Pollock et al. similarly found no detriment in outcomes when omitting GTV expansions in select p16-positive cases [17], reinforcing the concept that microscopic disease spread in HPV-associated oropharyngeal cancer may be effectively managed by elective-dose coverage or may be inherently limited. The current study provides detailed dosimetric confirmation that further margin reduction (from 5 mm to 0 mm) can translate into meaningful normal tissue sparing, which prior clinical outcome studies had implied but not explicitly quantified.

The degree of dysphagia risk reduction suggested by the Dutch NTCP modes aligns with the improvements seen in trials that took alternative approaches to spare swallowing structures. The DARS trial (Nutting et al.) did not alter CTV margins but instead selectively reduced the dose of the pharyngeal constrictors outside the tumour volume, achieving a 7.5 Gy lower mean constrictor dose. The margin-less approach in this study achieved a comparable reduction in mean PCM dose (~4–5 Gy), suggesting it might realise similar benefits in patient-reported outcomes [3].

When comparing the NTCP reduction achieved with margin-less planning to that of proton therapy, it is notable that the estimated dysphagia gain is of similar magnitude. In the pilot study of DAHANCA35, Hansen et al. reported a 5.3% reduction in dysphagia risk using clinical proton plans, which closely matches the 6.0% reduction observed with margin-less photon plans [18]. This suggests that omitting the CTV1 margin may offer a benefit comparable to proton therapy. Importantly, combining margin-less photon planning with proton therapy could potentially lead to an even greater NTCP reduction. The current estimate of ΔNTCP could be even high for patients with baseline dysphagia as the difference will increase slightly.

Another relevant study by Al-Mamgani et al. systematically reduced the GTV-to-CTV margin from 10 to 6 mm. They documented significant declines in both acute and late toxicities, including dysphagia and xerostomia, with no compromise in 2-year loco-regional control. They observed that feeding tube dependency at 6 months dropped from 15 to 6% with margin reduction [19, 20]. In the DAHANCA studies feeding tube dependency is often not used as an objective end point, as the dependency relies heavily on the proactive action of healthcare professionals, as some patients may continue using a feeding tube out of habit or convenience despite functional swallowing ability.

The prospect of completely omitting the high-risk CTV margin is intriguing, potentially for HPV-positive oropharyngeal cancers, which are typically radiosensitive and often occur in younger patients where long-term QoL matters greatly. By decreasing doses to swallowing muscles and salivary glands, this approach could reduce the incidence of chronic dysphagia, feeding tube dependence, and severe xerostomia – late effects that currently affect a significant subset of survivors [1]. Importantly, these benefits were achieved without any sophisticated planning techniques beyond what is standard. The study utilised a commercial auto-planning tool to ensure consistency; however, the concept is general, as any planning system can create these plans, as they are simply the result of smaller targets. In fact, planners might find it easier to meet stringent OAR constraints when the targets are smaller, as seen by the many significant dose reductions. The flip side is ensuring tumour coverage: with no margin. One must have high confidence in GTV delineation. Advanced imaging (MRI, PET) and perhaps, biologic imaging may help delineate GTVs more accurately, making the omission of margins safer [21]. The current study plans used a 3 mm PTV to ensure coverage for setup error; however, such a small PTV margin requires high-quality immobilisation and daily IGRT and 6D setup corrections [22].

There are several limitations of this study. First, it is a dosimetric planning study on a relatively small sample (*N* = 30) without clinical outcome data. While predicting toxicity reductions based on dose differences and NTCP models, the true impact on patient symptoms can only be confirmed in prospective trials. Second, the planning approach, which maintains coverage by delivering an elective dose to the 5 mm shell around the GTV, essentially treating it as CTV2, has not been tested prospectively. Tumours with suspected microscopic spread just beyond the GTV might require a compromise approach. Nonetheless, given the retrospective evidence, even cases with 0 mm margins did not fare worse [17]. Another limitation is that the study did not explicitly analyse acute toxicity endpoints. It is likely that acute mucositis and acute dysphagia, which are driven by doses to the oral cavity, pharynx, etc., would also be mitigated due to the OAR dose reduction. Less acute mucositis may have short-term benefits, such as improved treatment tolerance and fewer breaks. Finally, the use of auto-planning means these plans are optimised to a high degree; in less controlled settings, actual clinical plans might show more variability. The fact that only minimal manual adjustments were needed indicates feasibility, but centres without automated planning could still reproduce these results with careful planning [23].

The promising dosimetric results of this margin-elimination strategy justify further clinical evaluation. A logical next step would be a prospective trial for patients with favourable head and neck cancers, randomising between standard (5 mm margin) and margin-less target delineations as a non-inferior trial, with secondary endpoints of toxicity and QoL. Such a trial is being set up as a new DAHANCA trial randomising approximately 1,600 patients.

In conclusion, in the RT of oropharyngeal cancer, the traditional 5 mm expansion of GTV to CTV1 can be omitted in an auto-planning framework, resulting in markedly lower doses to normal structures. The retrospective planning analysis of 30 patients shows that using GTV = CTV1 (0 mm margin) achieves comparable target coverage to standard plans, while reducing mean doses to pharyngeal constrictors by 4–5 Gy and to major salivary glands by 2–5 Gy. These reductions are predicted to significantly lessen the risk of late dysphagia. The findings corroborate emerging clinical evidence that smaller high-dose volumes do not compromise tumour control but can improve tolerability. While clinical validation is required, the study provides a strong rationale for trials investigating margin-less target delineation in favourable head and neck cancer patients.

## Supplementary Material



## Data Availability

The data that support the findings of this study are available from the corresponding author upon reasonable request. Due to patient privacy regulations, individual treatment plans and imaging data cannot be made publicly available. However, aggregated dose-volume data for organs at risk (as presented in Tables 1 and 2) and plan quality metrics can be provided on request. All data sharing will comply with GDPR and institutional policies.
