# Supplementary material for: Eliminating the high-risk CTV1 margin in DAHANCA oropharyngeal radiotherapy: Dosimetric impact on dysphagia and organ-at-risk doses
Source: Acta Oncol. 2025 Sep 11;64:44049. doi: 10.2340/1651-226X.2025.44049 (PMC12439217; doi:10.2340/1651-226X.2025.44049)
Supplement: Supplementary file 1 [file AO-64-44049-s1.pdf]

Supplementary material has been published as submitted. It has not been copyedited, or typeset by Acta Oncologica

Appendix:

Figure 1

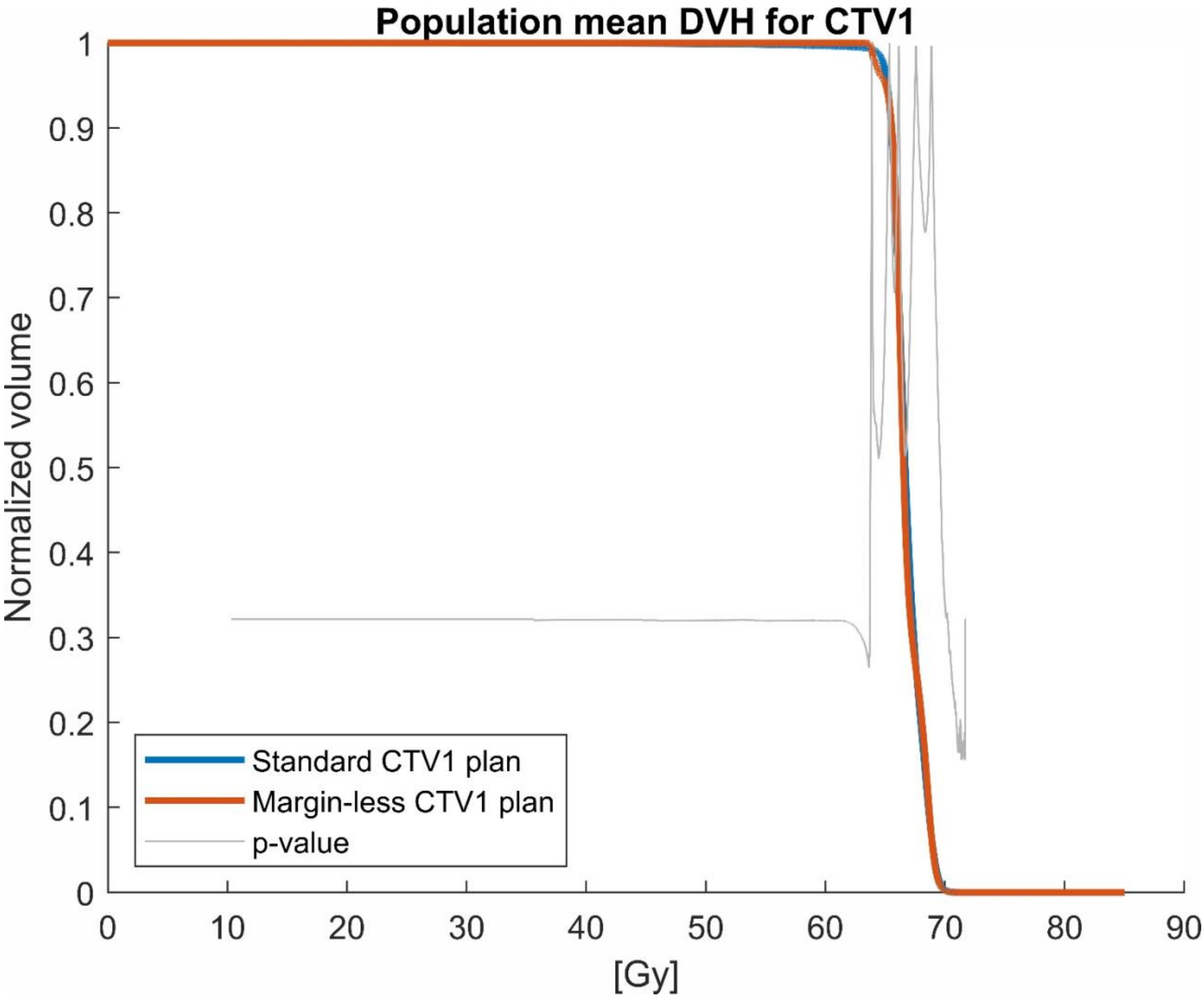

Population mean dose-volume histograms (DVHs) for selected target-adjacent structures comparing standard CTV1 plans (blue) and margin-less CTV1 plans (red). The margin-less strategy consistently reduces dose to surrounding tissues while maintaining target coverage. The gray curve represents dose-bin-wise p-values indicating statistically significant differences between the two plan types across the dose range.

Figure 2

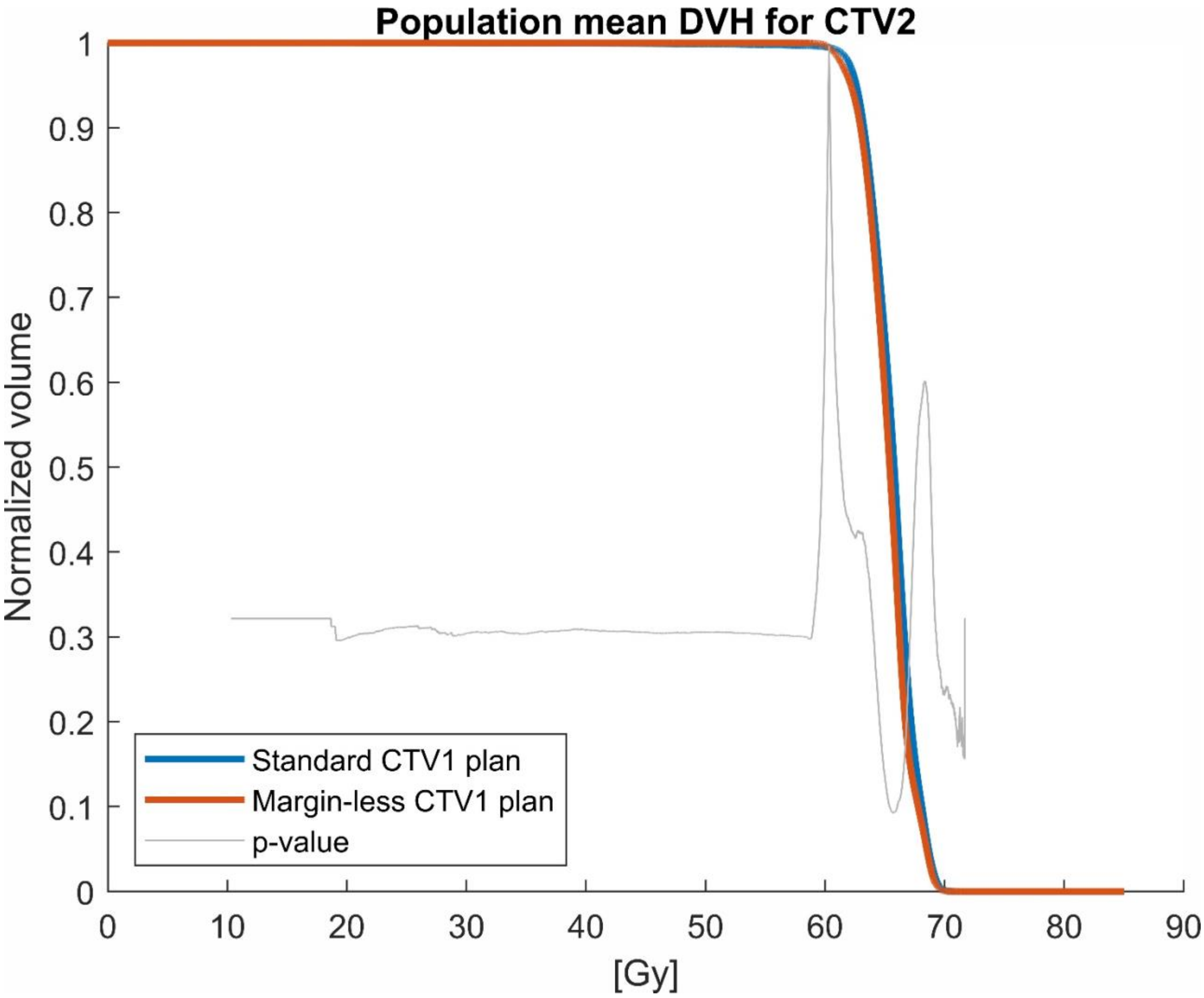

Population mean dose-volume histograms (DVHs) for selected target-adjacent structures comparing standard CTV1 plans (blue) and margin-less CTV1 plans (red). The margin-less strategy consistently reduces dose to surrounding tissues while maintaining target coverage. The gray curve represents dose-bin-wise p-values indicating statistically significant differences between the two plan types across the dose range.

Figure 3

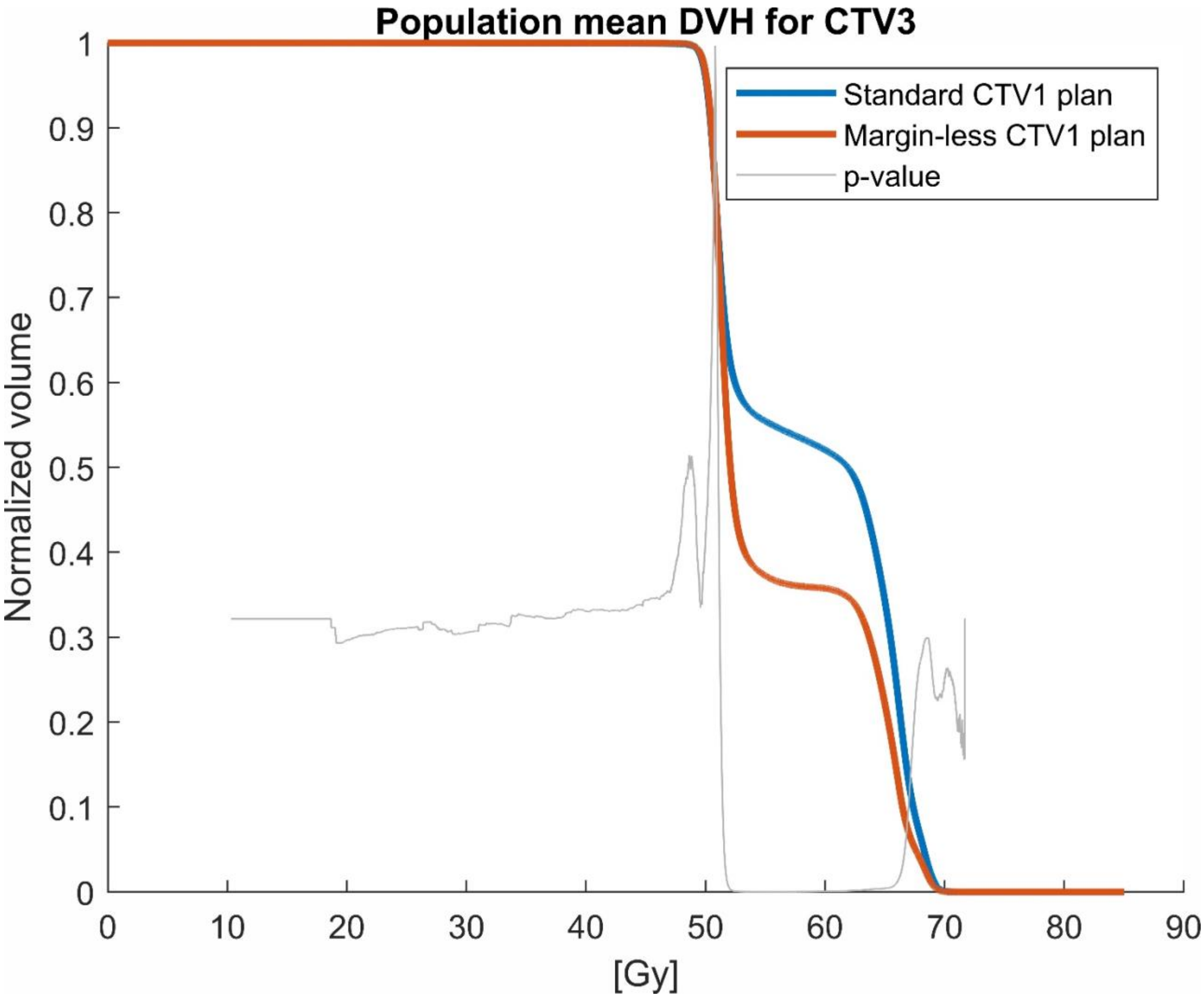

Population mean dose-volume histograms (DVHs) for selected target-adjacent structures comparing standard CTV1 plans (blue) and margin-less CTV1 plans (red). The margin-less strategy consistently reduces dose to surrounding tissues while maintaining target coverage. The gray curve represents dose-bin-wise p-values indicating statistically significant differences between the two plan types across the dose range.

Figure 4

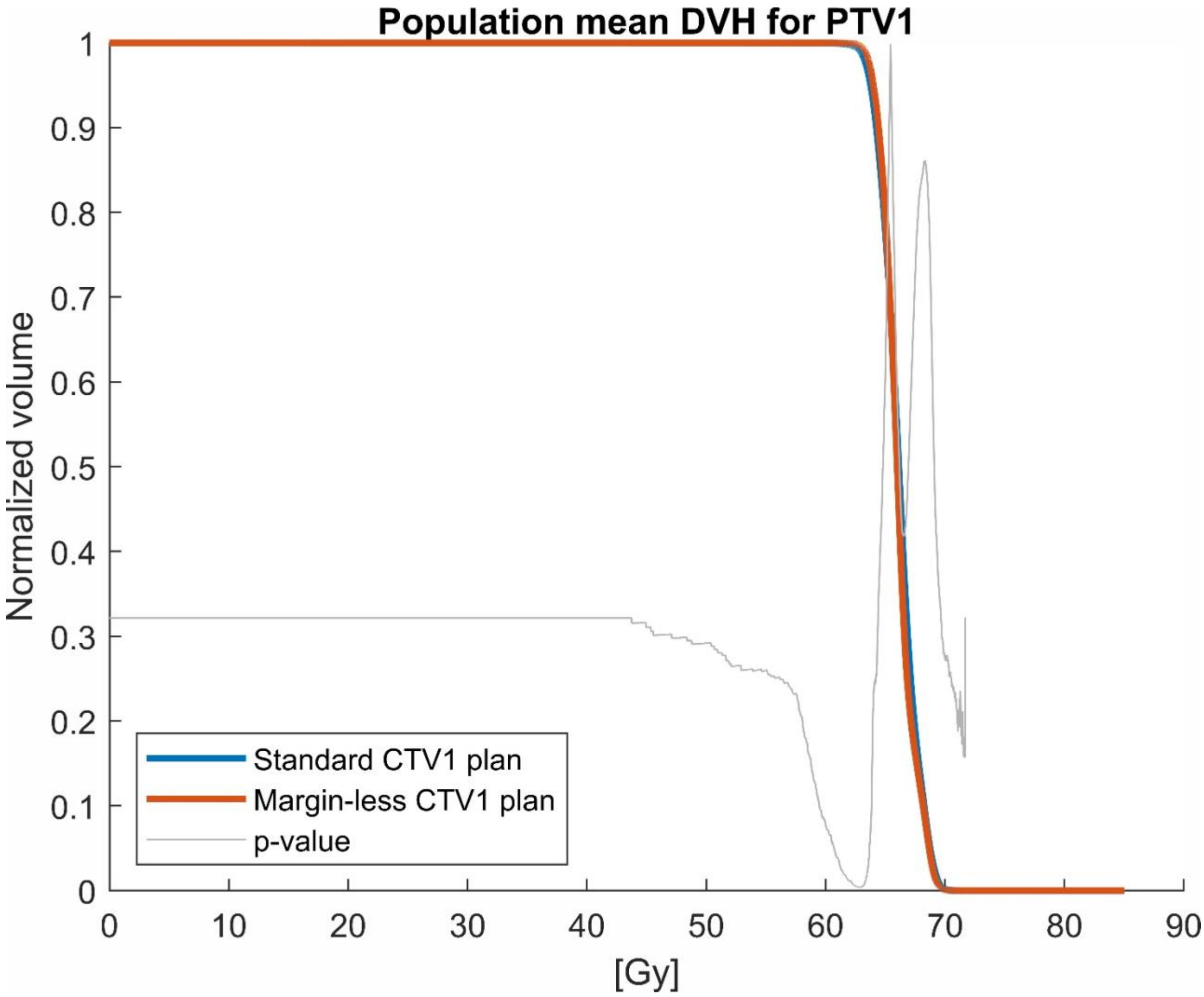

Population mean dose-volume histograms (DVHs) for selected target-adjacent structures comparing standard CTV1 plans (blue) and margin-less CTV1 plans (red). The margin-less strategy consistently reduces dose to surrounding tissues while maintaining target coverage. The gray curve represents dose-bin-wise p-values indicating statistically significant differences between the two plan types across the dose range.

Figure 5

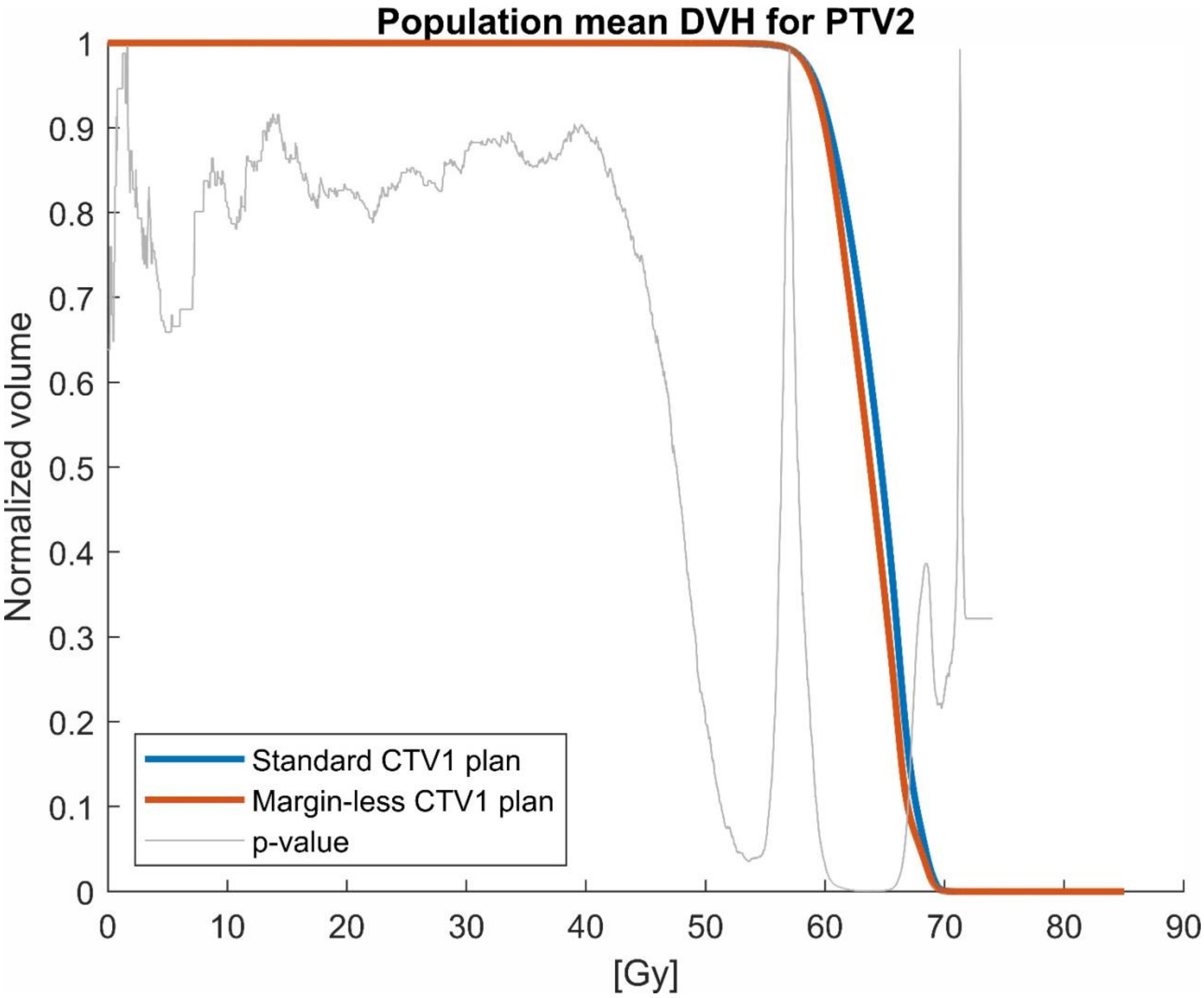

Population mean dose-volume histograms (DVHs) for selected target-adjacent structures comparing standard CTV1 plans (blue) and margin-less CTV1 plans (red). The margin-less strategy consistently reduces dose to surrounding tissues while maintaining target coverage. The gray curve represents dose-bin-wise p-values indicating statistically significant differences between the two plan types across the dose range.

Figure 6

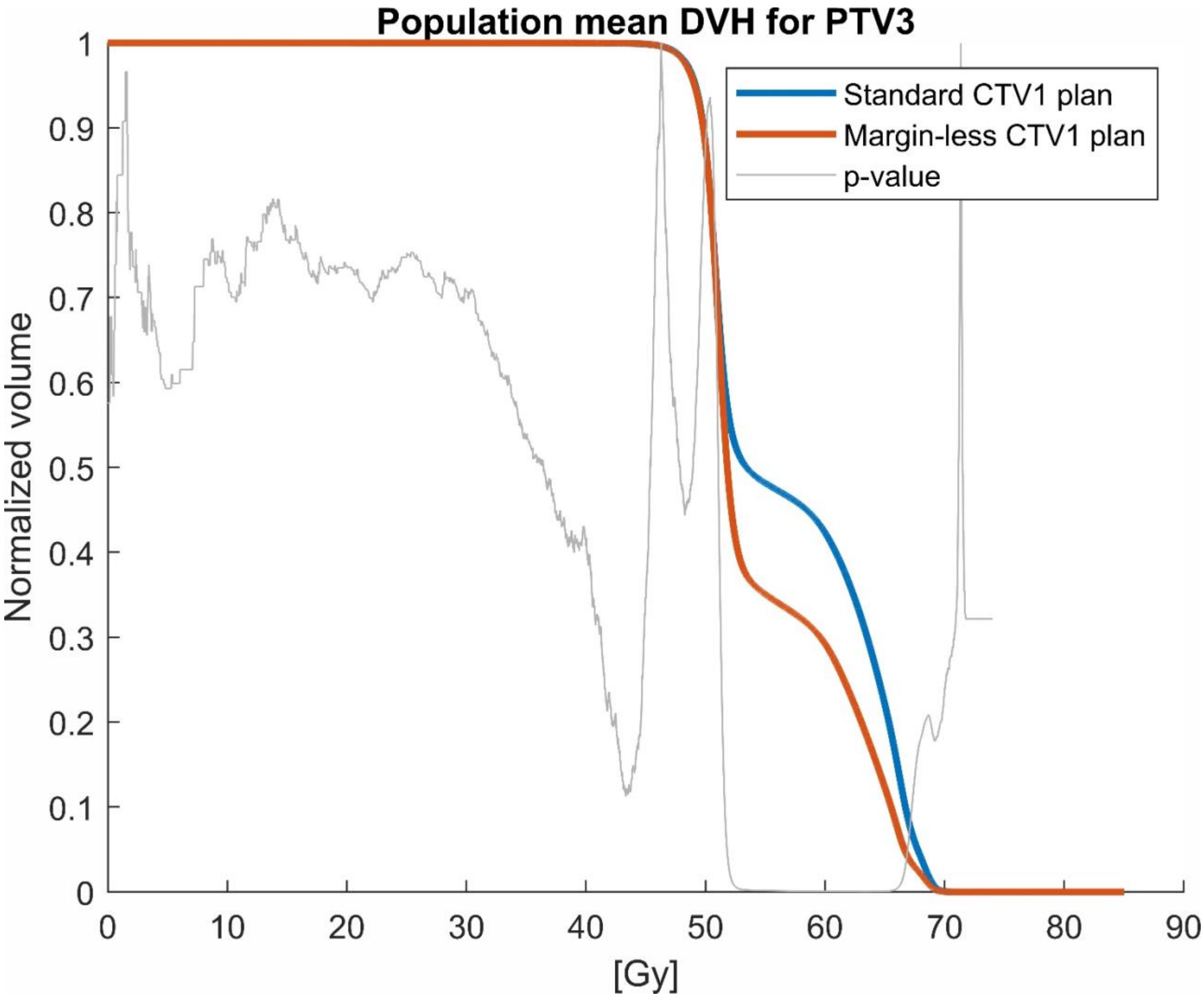

Population mean dose-volume histograms (DVHs) for selected target-adjacent structures comparing standard CTV1 plans (blue) and margin-less CTV1 plans (red). The margin-less strategy consistently reduces dose to surrounding tissues while maintaining target coverage. The gray curve represents dose-bin-wise p-values indicating statistically significant differences between the two plan types across the dose range.

Figure 7

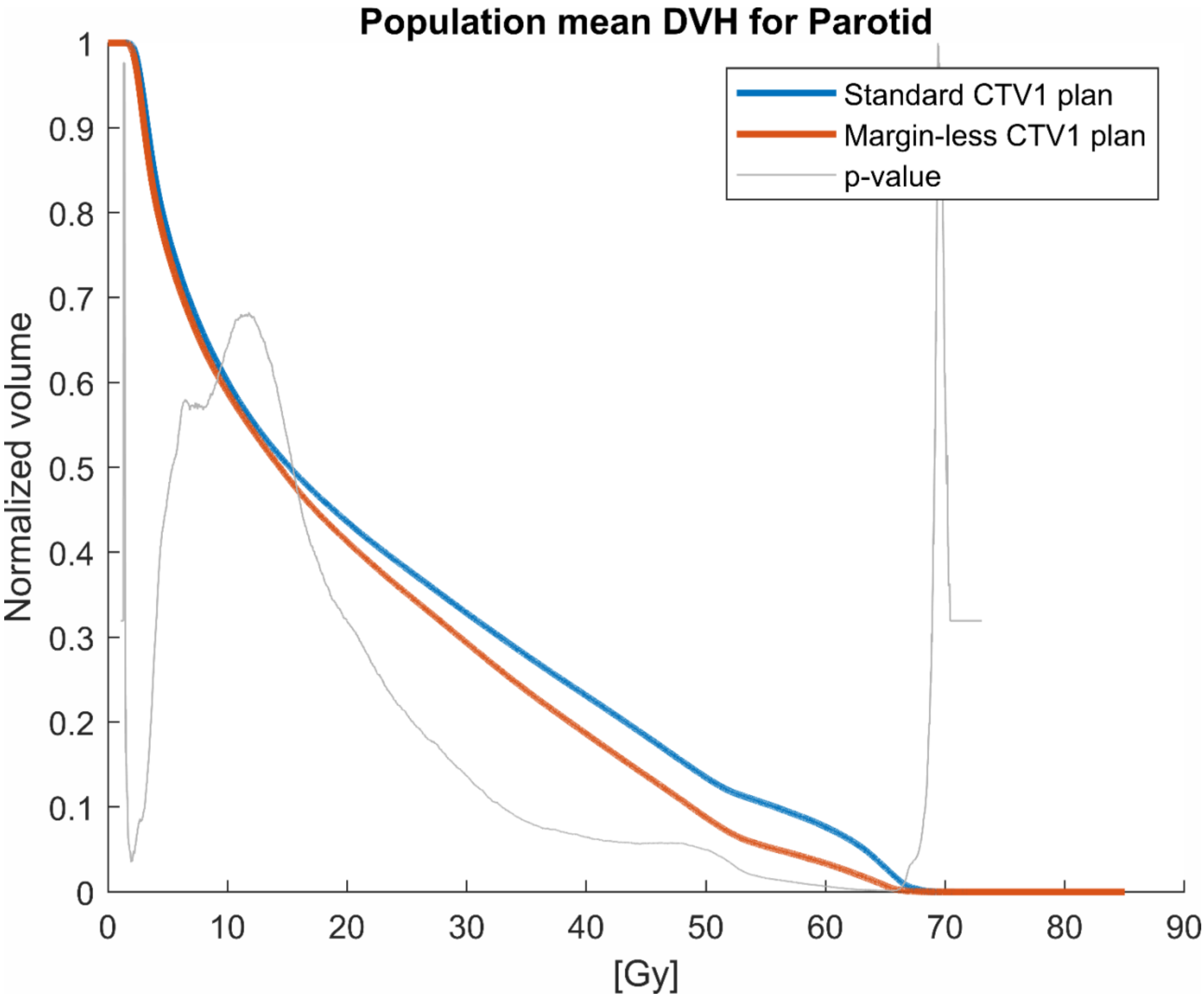

Population mean dose-volume histogram (DVH) for the [OAR name] comparing standard CTV1 plans (blue) and margin-less CTV1 plans (red). Dose-bin-wise p-values (gray) indicate statistically significant differences across relevant portions of the dose distribution.

Figure 8

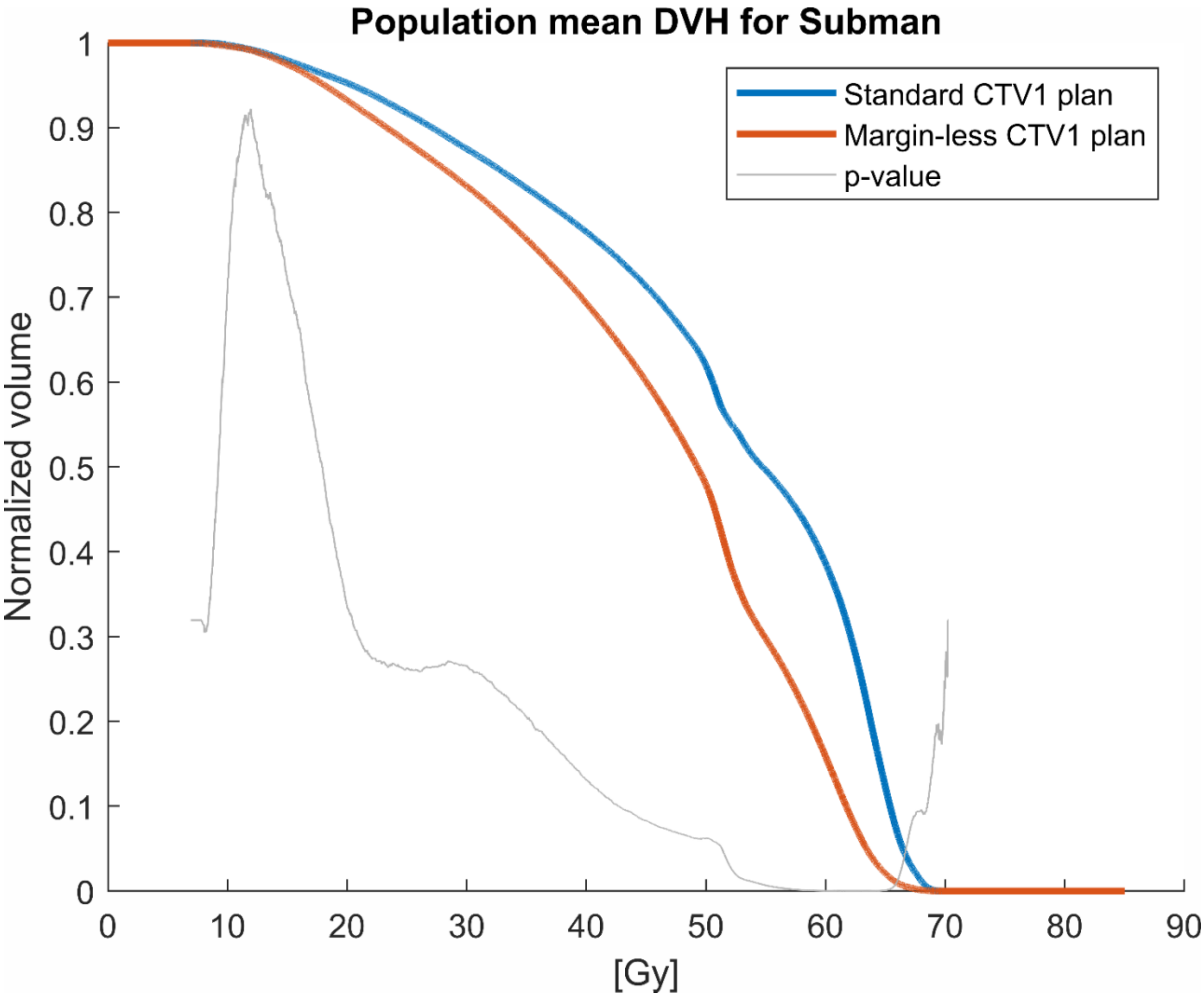

Population mean dose-volume histogram (DVH) for the [OAR name] comparing standard CTV1 plans (blue) and margin-less CTV1 plans (red). Dose-bin-wise p-values (gray) indicate statistically significant differences across relevant portions of the dose distribution.

Figure 9

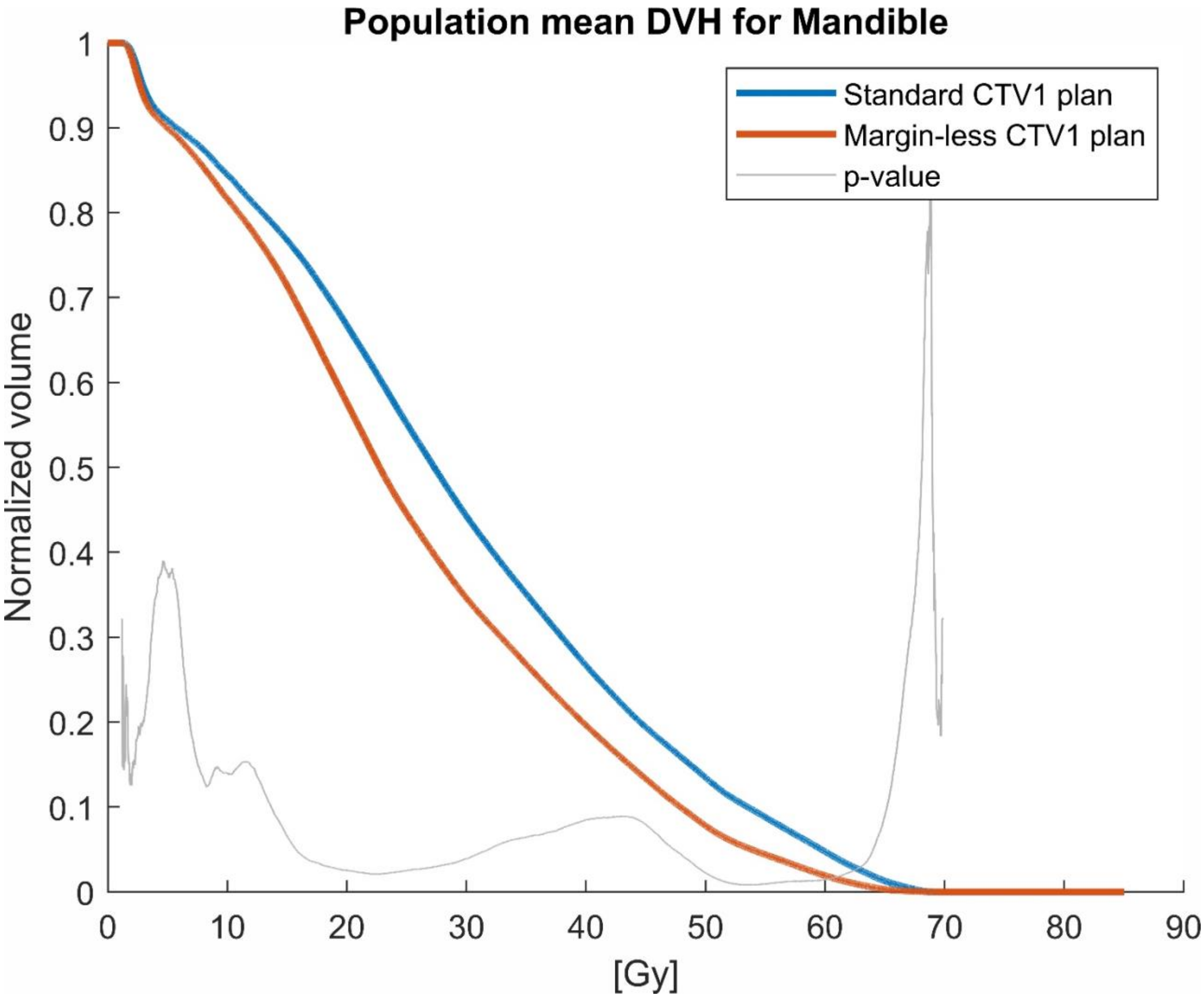

Population mean dose-volume histogram (DVH) for the [OAR name] comparing standard CTV1 plans (blue) and margin-less CTV1 plans (red). Dose-bin-wise p-values (gray) indicate statistically significant differences across relevant portions of the dose distribution.

Figure 10

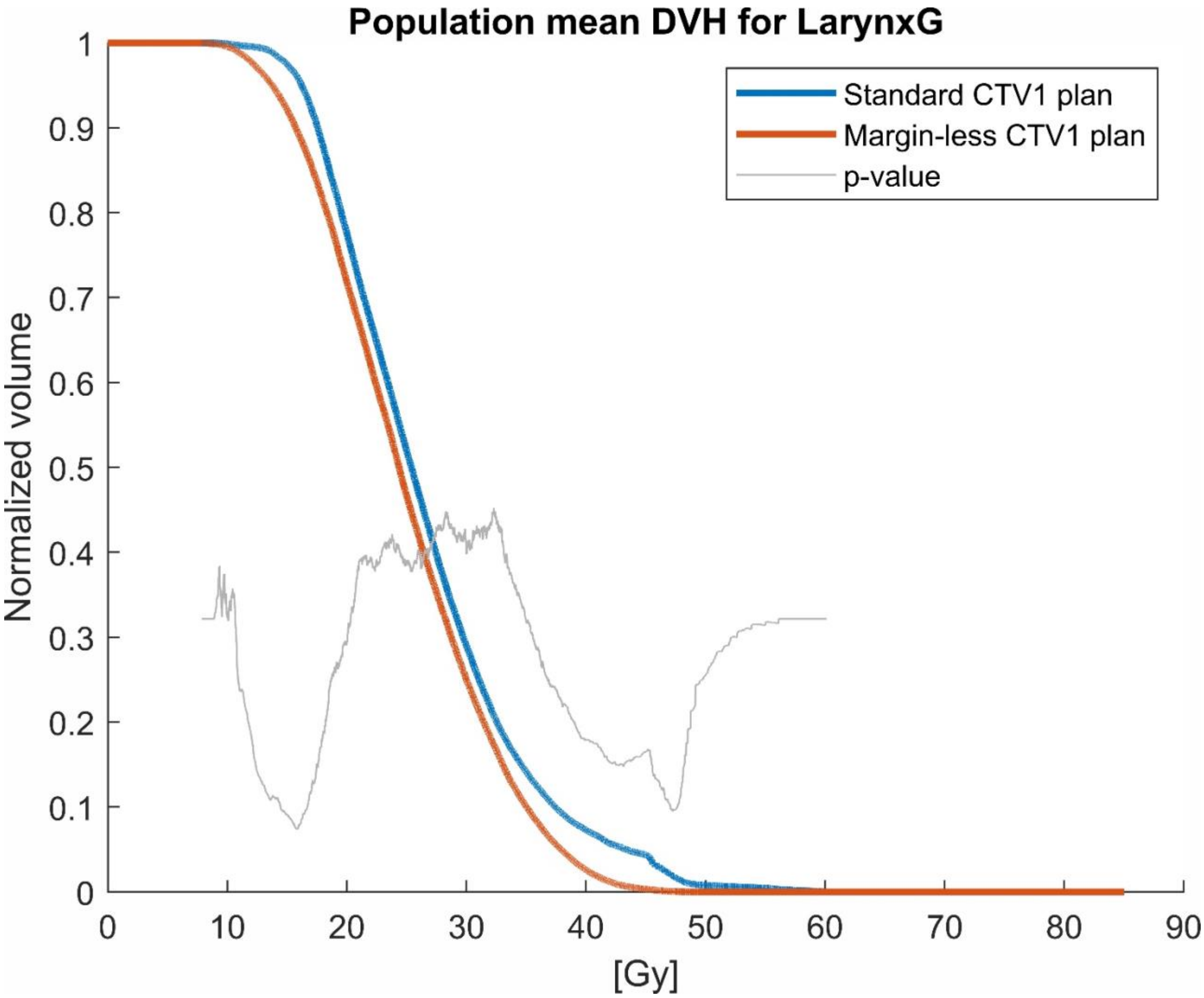

Population mean dose-volume histogram (DVH) for the [OAR name] comparing standard CTV1 plans (blue) and margin-less CTV1 plans (red). Dose-bin-wise p-values (gray) indicate statistically significant differences across relevant portions of the dose distribution.

Figure 11

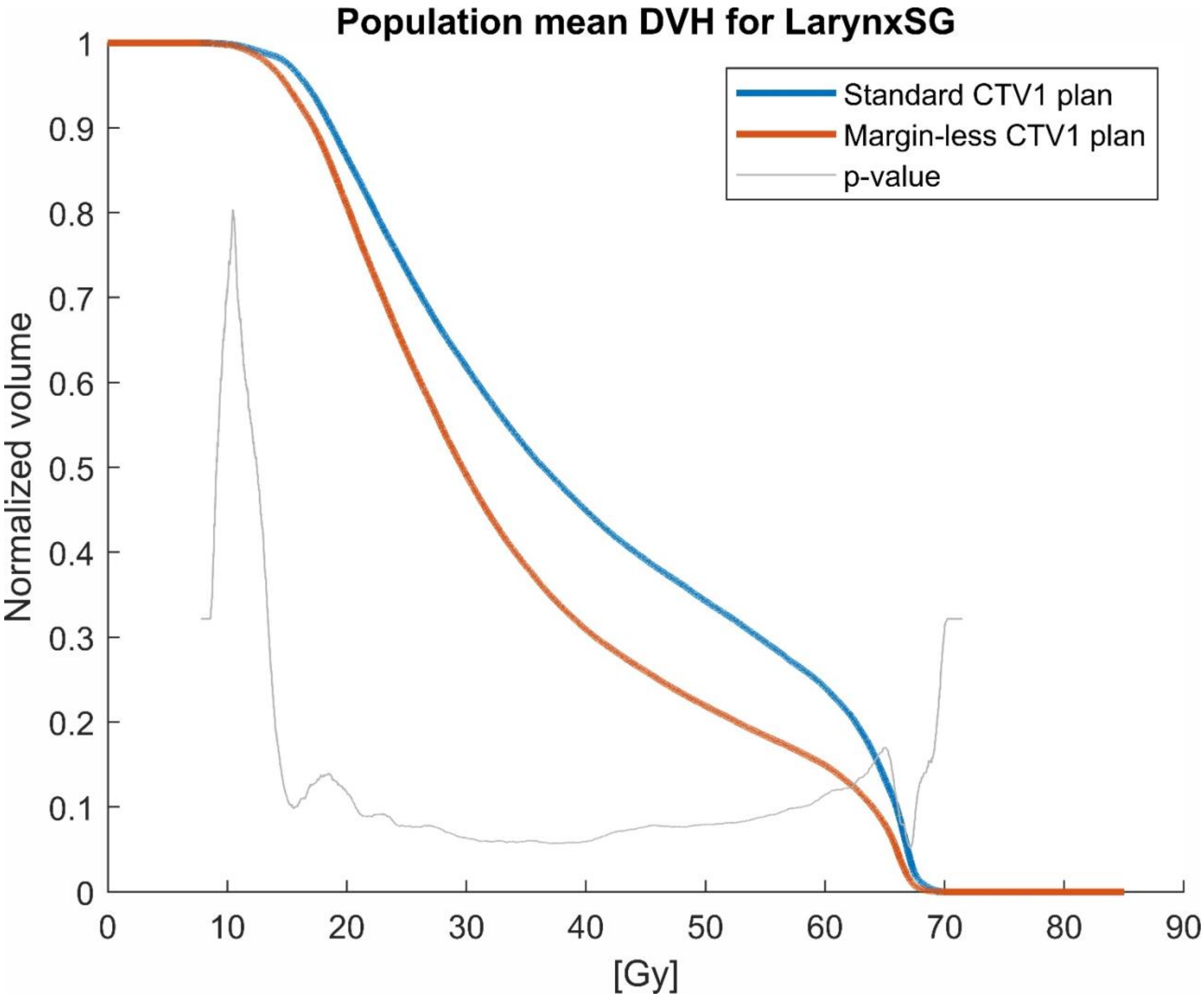

Population mean dose-volume histogram (DVH) for the [OAR name] comparing standard CTV1 plans (blue) and margin-less CTV1 plans (red). Dose-bin-wise p-values (gray) indicate statistically significant differences across relevant portions of the dose distribution.

Figure 12

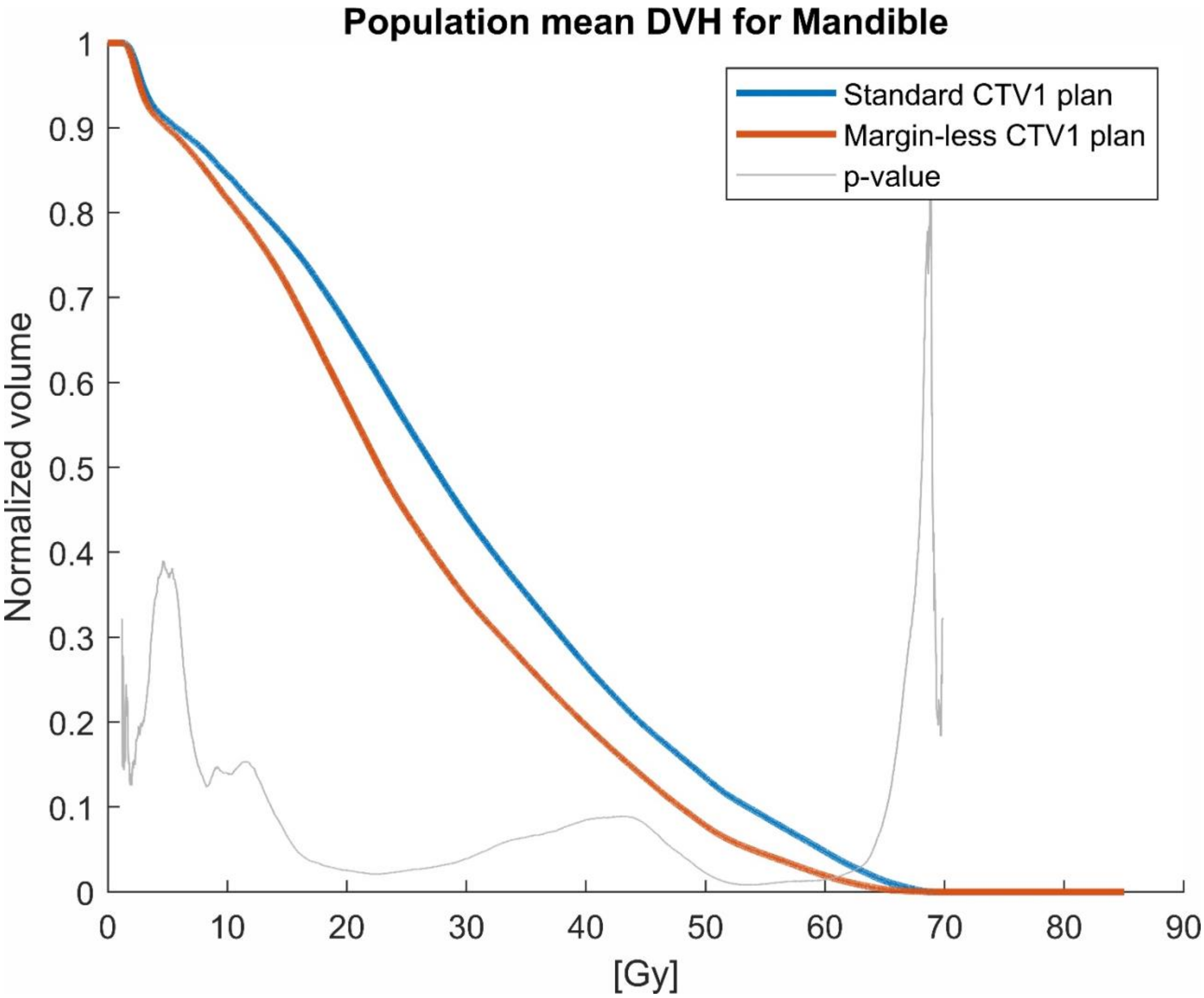

Population mean dose-volume histogram (DVH) for the [OAR name] comparing standard CTV1 plans (blue) and margin-less CTV1 plans (red). Dose-bin-wise p-values (gray) indicate statistically significant differences across relevant portions of the dose distribution.

Figure 13

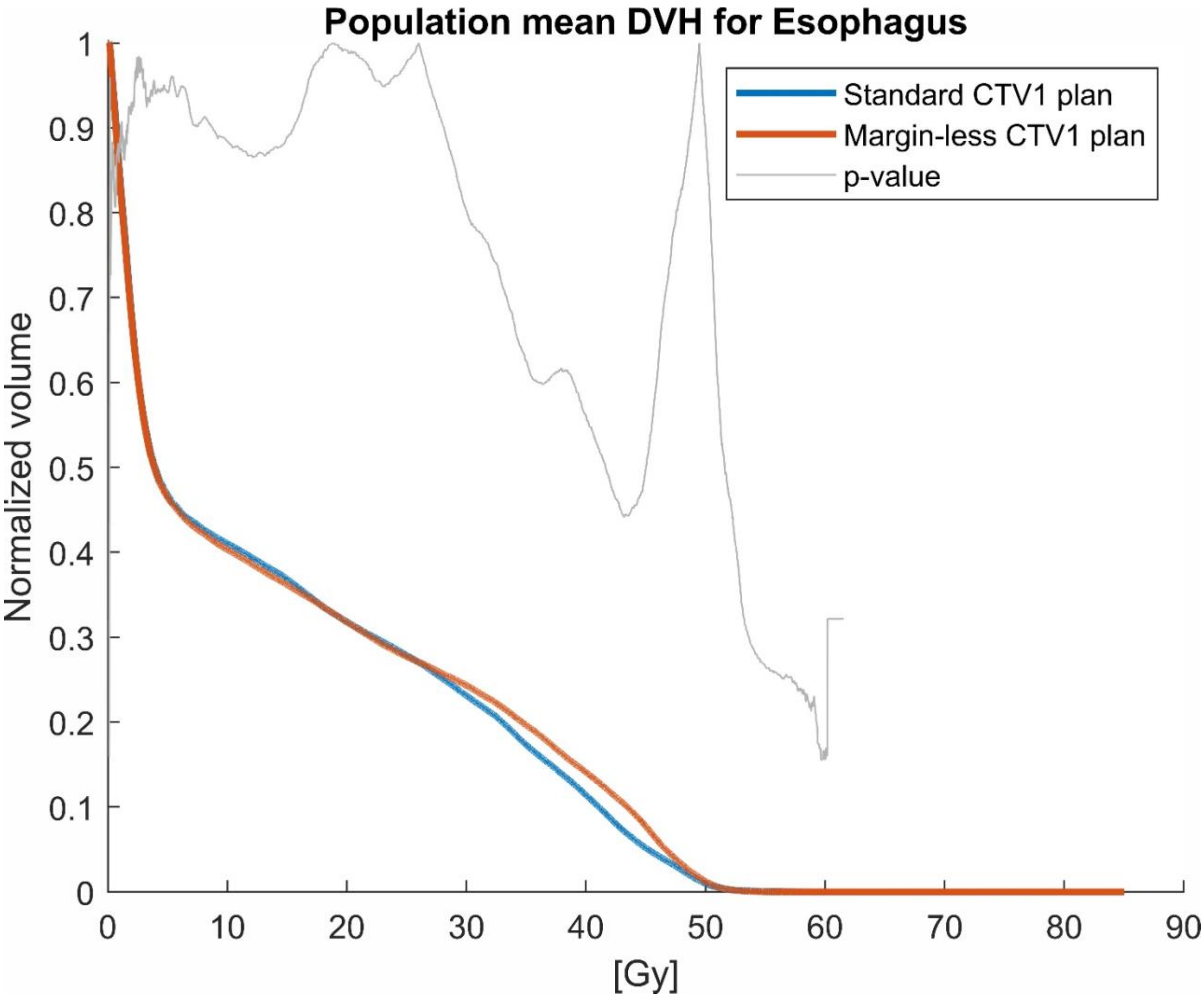

Population mean dose-volume histogram (DVH) for the [OAR name] comparing standard CTV1 plans (blue) and margin-less CTV1 plans (red). Dose-bin-wise p-values (gray) indicate statistically significant differences across relevant portions of the dose distribution.

Figure 14

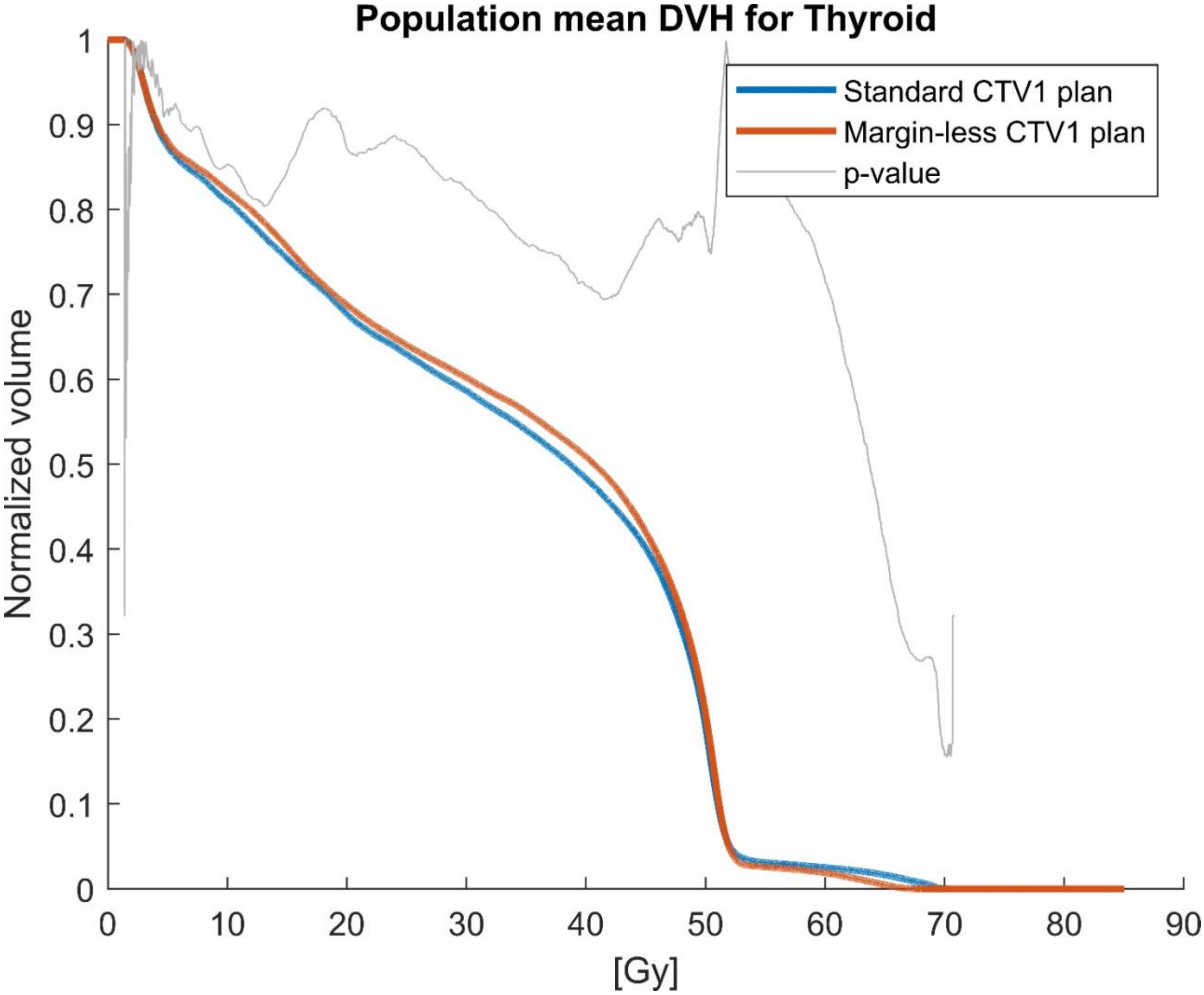

Population mean dose-volume histogram (DVH) for the [OAR name] comparing standard CTV1 plans (blue) and margin-less CTV1 plans (red). Dose-bin-wise p-values (gray) indicate statistically significant differences across relevant portions of the dose distribution.

Figure 15

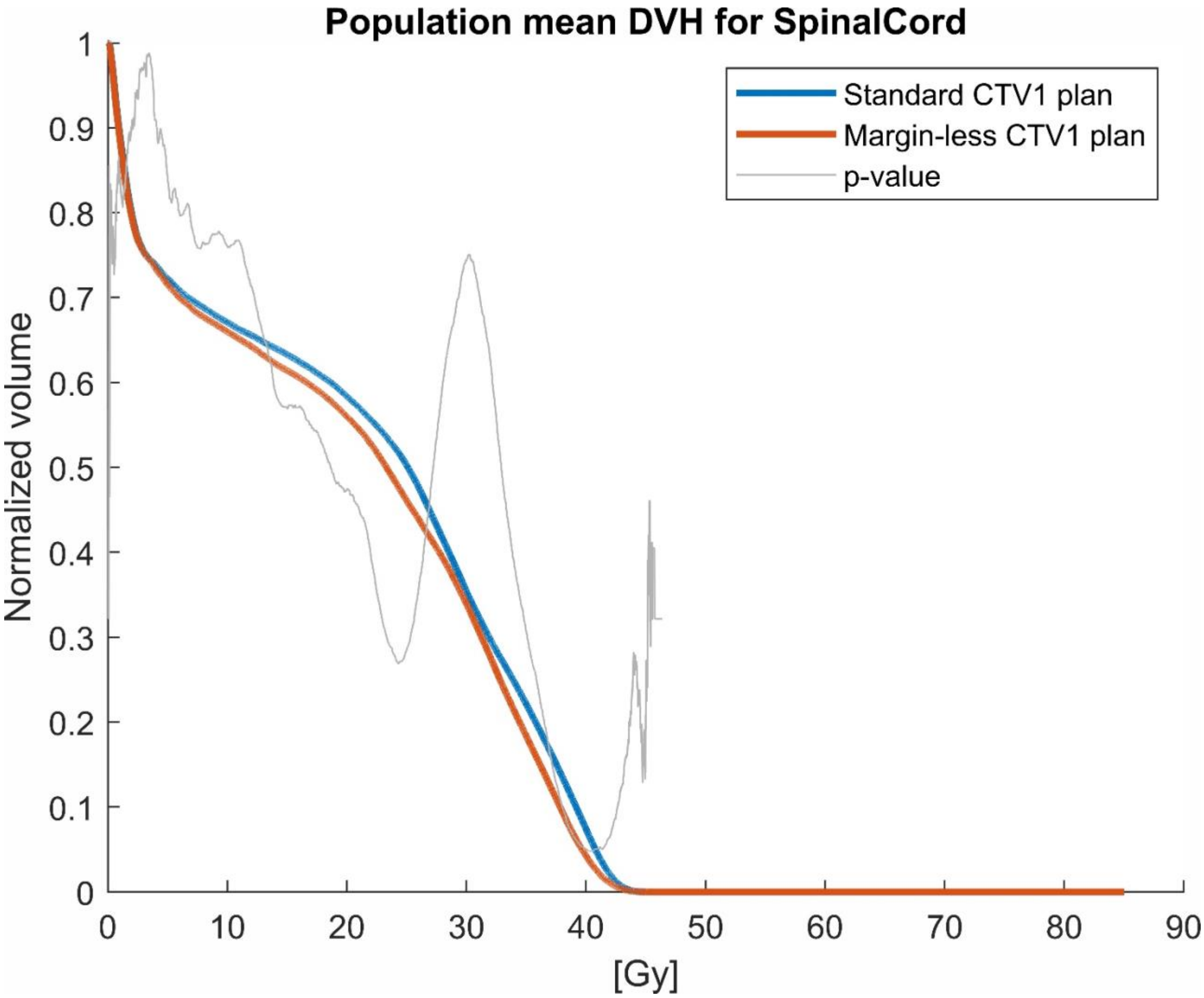

Population mean dose-volume histogram (DVH) for the [OAR name] comparing standard CTV1 plans (blue) and margin-less CTV1 plans (red). Dose-bin-wise p-values (gray) indicate statistically significant differences across relevant portions of the dose distribution.

Figure 16

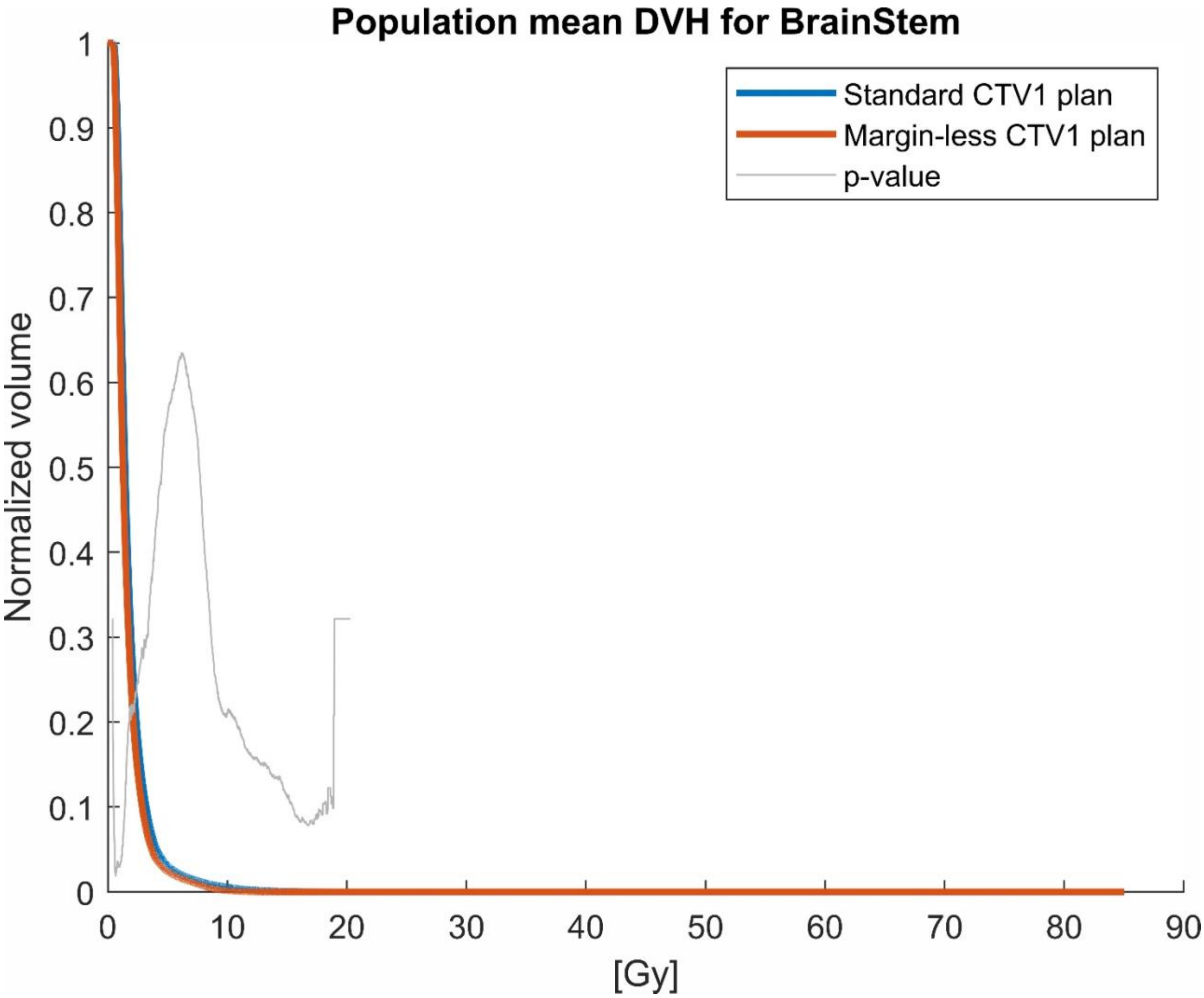

Population mean dose-volume histogram (DVH) for the [OAR name] comparing standard CTV1 plans (blue) and margin-less CTV1 plans (red). Dose-bin-wise p-values (gray) indicate statistically significant differences across relevant portions of the dose distribution.
